# Characterization of rhinovirus C from a 4-year-old boy with acute onset dilated cardiomyopathy in Jakarta, Indonesia

**DOI:** 10.1099/jmmcr.0.005139

**Published:** 2018-02-01

**Authors:** Ageng Wiyatno, E. S. Zul Febrianti, Aghnianditya Kresno Dewantari, Khin Saw Myint, Dodi Safari, Nikmah Salamia Idris

**Affiliations:** ^1^​ Eijkman Institute for Molecular Biology, Jakarta, Indonesia; ^2^​ Cardiology Division, Department of Child Health, Faculty of Medicine Universitas Indonesia/Cipto Mangunkusumo National General Hospital, Jakarta, Indonesia

**Keywords:** rhinovirus C, myocarditis, cardiomyopathy, Indonesia

## Abstract

**Introduction:**

Myocarditis, inflammation of the heart muscle, can be caused by infections, autoimmune disease or exposure to toxins. The major cause of myocarditis in the paediatric population is viral infection, including coxsackievirus B3, adenovirus, herpesvirus, parvovirus, influenza A and B, and hepatitis. Here, we report the detection of rhinovirus C in a boy with a clinical presentation of myocarditis, suggesting a possible causative role of this virus in this case.

**Case presentation:**

A previously well 4.5-year-old boy presented with increasing breathlessness for a week prior to admission. He also had upper respiratory tract infection a few days before the event. An echocardiogram revealed severe left ventricle (LV) systolic dysfunction with dilation of the LV. RNA was extracted from serum and two nasal swabs, and tested with conventional PCR at the family level for viruses including enterovirus, dengue, chikungunya, influenza, herpesvirus, paramyxovirus and coronavirus. Further characterization of the enterovirus group was carried out using PCR with primers targeting the VP4/VP2 gene, followed by sequencing. Molecular tests showed the presence of rhinovirus C genetic material in both serum and swab samples. Phylogenetic analysis of the VP4/VP2 region showed 96–97 % similarity with the closest strain isolated in Ulaanbaatar (Mongolia) and Japan in 2012.

**Conclusion:**

We report the possible association of rhinovirus C and myocarditis in a child presenting with acute onset of dilated cardiomyopathy.

## Introduction

Dilated cardiomyopathy (DCM) is one of the most common and devastating heart diseases in children. Children with DCM mostly have to be on chronic heart failure medications and often progress into severe heart failure, requiring heart transplantation with or without a bridging mechanical heart. About half of the children were reported to have an identified cause of DCM [1] compared to 9–16 % in adults [2, 3]. One of the most frequent causes of DCM in young children is myocarditis or inflammation of the heart muscle, which can be caused by various conditions, including viral or bacterial pathogens, autoimmune disease or exposure to toxins [4]. The most common viral causes of myocarditis in the paediatric population include coxsackievirus B3, adenovirus, herpesvirus, parvovirus, influenza A and B, and hepatitis viruses [5–8]. Myocarditis is also detected during post mortem in 2–46 % cases of sudden death in young people [2, 3].

The gold standard for confirming viral aetiology is virus detection from heart muscle biopsy sample, an invasive method that carries a substantial risk, particularly if the child is unwell or in severe heart failure [9–11]. Rhinovirus, a member of the genus *Enterovirus*, family *Picornaviridae*, is the leading cause of upper respiratory infection in the world; it is the most frequently detected virus in human infection worldwide [12, 13]. However, lower respiratory tract infections in infants and children associated with severe clinical manifestations caused by rhinovirus could be underestimated due to lack of screening. Predisposing factors and underlying conditions are known to contribute to rhinovirus-associated severe respiratory diseases [14]. One study reported the possible link between rhinoviruses and congenital heart disease [14]; however, the causal role of rhinoviruses in cardiac conditions remains elusive. The genome of the virus is a positive sense ssRNA with approximately 7200 bp [15], and classified at least into three groups of rhinoviruses: A, B and C. Rhinovirus C, detected in 2006, was classified as a new species in 2010 [16]. This group was known to be associated with more severe illness than rhinovirus A and B, and resistant to commonly used antiviral treatments for rhinovirus [17]. A number of studies have reported the association of rhinovirus C with increased wheezing, asthma exacerbations and lower respiratory tract infection in children [7]. The virus was also detected in the bronchoalveolar lavage specimen, pericardial fluid, plasma and stools of a 14-year-old patient with pericarditis [18]. However, the association of rhinovirus C with DCM or myocarditis is not well documented. Currently, there are about 55 different types of human rhinovirus C, with 13 % variability in VP1 and at least 10 % variability in VP4/VP2. Vaccine and specific viral therapies for rhinoviruses are not currently available, and treatment is primarily supportive [15]. Here, we report the viraemic detection of rhinovirus C in a 4-year-old boy with acute onset DCM, suggesting a likely possible causative role of this virus in severe cardiac disease.

## Case report

A previously well four-and-a-half year-old boy presented with an upper respiratory tract infection, loss of appetite, fatigue and increasing breathlessness in the past 1 week before admission. There was no history of chest pain, cyanosis, joint pain or swelling. Predisposing factors, such as congenital cardiac disease, were excluded by review of the patient’s medical records. The patient received treatment in a primary care centre before being admitted to hospital. On examination, he was tachypnoeic and pale, with clammy extremities. His heart rate was 90 beats min^−1^, respiratory rate 30 breaths min^−1^, blood pressure 100/60 mmHg and temperature 37 °C. There was a mild face and lower limb oedema. On cardiac examination, there was decreased cardiac impulse on palpation. Heart sounds were muffled and no murmur was heard. His liver was palpable 3 cm below the costal angle. Pulses were palpable and equal in all four extremities. Laboratory examinations revealed a normal haemoglobin level of 132 g l^−1^ with a slightly increased white blood cell count (15 900 cells mm^−3^), whereas the platelet count was within normal limits (342 000 platelets mm^−3^). Unfortunately, cardiac markers such as troponin, creatinine kinase (CK) and its MB isoenzyme (CK-MB) were not tested due to limited resources. Chest X-ray showed cardiomegaly with congested lungs. Electrocardiography (ECG) revealed a sinus rhythm with a heart rate of 136 beats min^−1^. There was a normal P wave with slightly prolonged PR interval (>0.2 s). The QRS axis was normal and a possible left ventricular hypertrophy. There was no obvious ST change. Informed consent for patient management was taken by the clinician in the hospital before examination.

## Diagnosis

Echocardiography demonstrated moderate-to-severe dilation of the left ventricle (LV), with moderate central mitral regurgitation and mild tricuspid regurgitation (estimated right ventricular systolic pressure = 30 mmHg + right atrial pressure). There was severely reduced LV systolic function (ejection fraction 18 % by Simpson method), whereas right ventricular function was preserved. There was no sign of LV non-compaction or coronary abnormality. Magnetic resonance imaging was not performed in this case. Biological samples, including blood and nasal swabs, were collected as part of the routine clinical diagnostic procedure at the hospital.

The serum and two nasal swabs collected from the patient were sent to the Eijkman Institute, Jakarta, Indonesia, for screening with comprehensive viral detection panels. The testing of depersonalized diagnostic specimens was approved by the Eijkman Institute Research Ethics Commission (number 66, 18 November 2013).

Viral RNA was obtained from 140 µl nasal swab in viral transport medium and serum from the patient using a QIAamp viral RNA mini kit (Qiagen). cDNA synthesis was performed using a Go Taq reverse transcription system (Promega), following the manufacturer’s instructions. The cDNA was then used as a template for detection by *Enterovirus* genus-level PCR targeting the 5′ untranslated region (UTR), following a published report [19]. Additional screening for dengue, chikungunya, coxsackievirus, influenza, herpesvirus, paramyxovirus and coronavirus was also performed following our laboratory algorithm. The PCR product of the VP4/VP2 region was sequenced using Big Dye Terminator and Sanger sequencing for genotyping and phylogenetic analysis. The VP4/VP2 region sequence was analysed and compared with a selection of reference strains from the GenBank database using Geneious software R8 version 8.1 [20]. A phylogenetic tree was reconstructed on the basis of 433 nt fragments of the VP4/VP2 region applying the Kimura 2-parameter method with 1000 bootstraps of the neighbour-joining model as previously detailed.

**Fig. 1.**
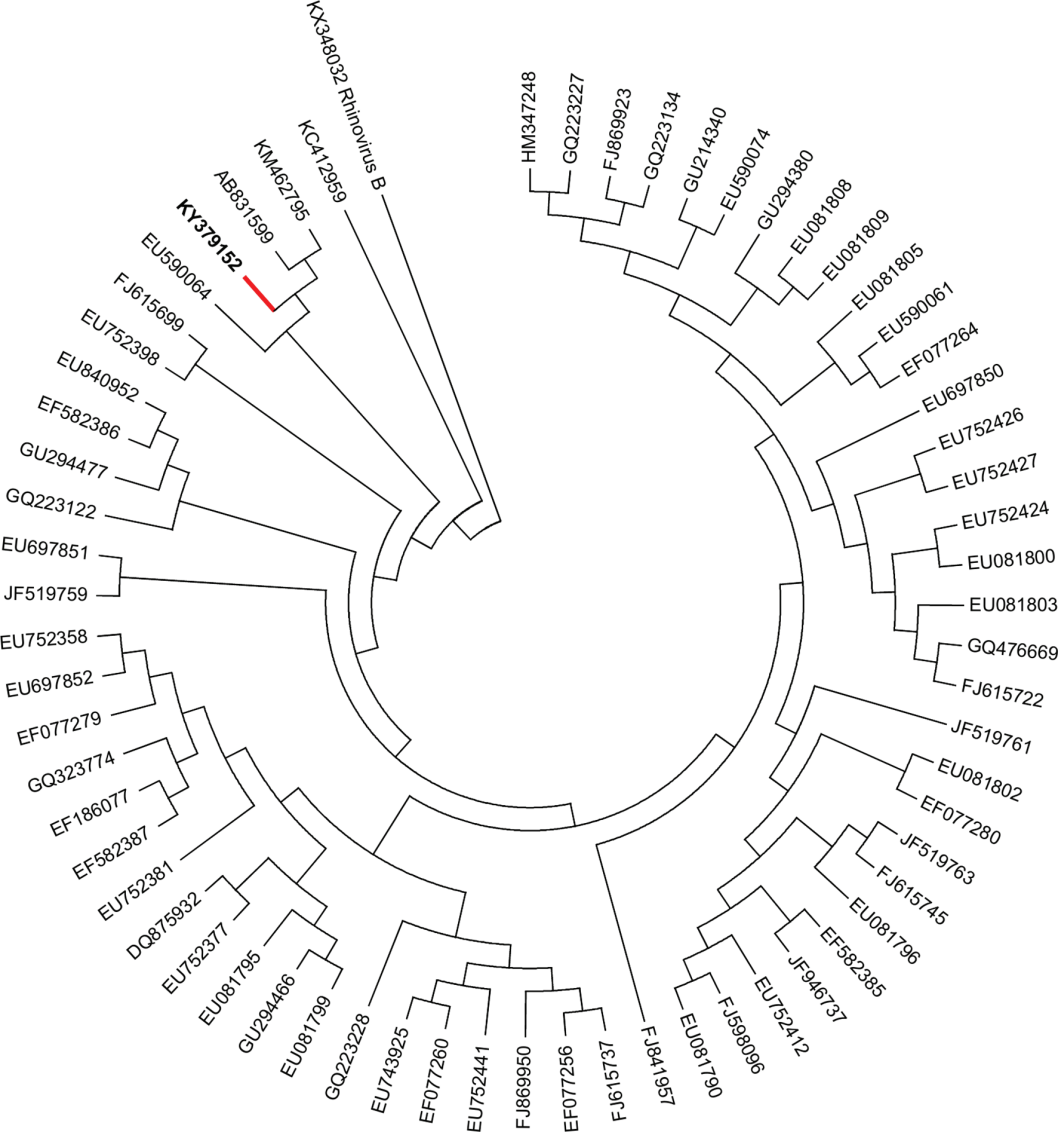
Maximum‐likelihood tree of the VP4/VP2-encoding region, showing the relationship of the Indonesian strain (red branch) with other strains of rhinovirus C. The sequence has similarity of only 97 % with the closest strain isolated in Ulaanbaatar (Mongolia) (KM462795) and Japan (AB831599) in 2012. The ML tree was reconstructed using Geneious R8 (HKY model and 1000 bootstraps), with rhinovirus B (GenBank accession number KX348042) as the outgroup.

PCR detection using an enterovirus group panel targeting the UTR was positive in both serum and nasal swab samples, indicating that the patient was viraemic during that period. Sequencing the 400 bp UTR indicated the presence of rhinovirus C genetic material. Further characterization of this Indonesian strain rhinovirus C was conducted using PCR with primers targeting the VP4/VP2 gene. The 390 bp VP4/VP2 sequence that was obtained and compared to an online nucleotide database using blastn (National Center for Biotechnology Information) showed 96–97 % similarity with the closest strain isolated in Ulaanbaatar (Mongolia) and Japan in 2012. Other infections associated with myocarditis, such as dengue, chikungunya, coxsackievirus, herpesvirus, paramyxovirus, coronavirus and influenza, were ruled out. Virus isolation was not attempted, as rhinovirus C could not be propagated in regular epithelial cells or immortalized cell lines [21]. The sequence has been submitted to GenBank with accession number KY379152 (Fig. 1).

## Treatment

The patient was treated with diuretics (furosemide, 20 mg 12 hourly; spironolactone, 12.5 mg 24 hourly), captopril (7.5 mg 12 hourly) and aspirin (80 mg 24 hourly).

## Outcome and follow-up

After 5 days of hospitalization, heart failure symptoms improved and the child was discharged. On the outpatient follow-up, his exercise tolerance improved; however, the patient still had residual left ventricular systolic dysfunction (ejection fraction 35–45 %, fraction shortening 20 %) and mild LV dilation at 6 months follow up.

## Discussion

Rhinovirus C, first identified a decade ago, is reportedly associated with mild infections in humans. However, with a 10–20 % prevalence in respiratory tract infections, rhinovirus C is widespread, contagious and genetically diverse [22]. We report here a possible unusual clinical manifestation of rhinovirus C in a child presenting with acute onset DCM in Jakarta, Indonesia.

Analysis on the VP4/VP2 region of the virus showed only 96–97 % similarity with the strain detected in Ulaanbaatar (Mongolia) and Japan in 2012; however, the circulation of this virus in Indonesia has not been reported. Rhinovirus C is known to undergo mutations at about 3.07×10^−3^ substitutions per site per year [22], which can give rise to widespread outbreaks and unusual manifestations. The association between rhinovirus C and DCM or myocarditis has never been reported previously.

Our study has several limitations. We could not confirm myocarditis, or specifically rhinovirus infection, as the cause for the acute onset DCM as we did not have the capacity to test the cardiac biomarkers nor to perform a cardiac biopsy. However, the clinical course of our patient suggested an acute onset cardiomyopathy, possibly related to myocarditis. Also, we were not able to exclude other possible causes of myocarditis, such as adenovirus, parvovirus B19 and hepatitis C virus, or autoimmune disease and environmental toxins, although the clinical picture was not consistent with the latter two causes. Review of the patient’s medical reports showed no predisposing or underlying conditions that might play a role in the pathogenesis as documented with severe respiratory infections.

In conclusion, we report the possible association of rhinovirus C and acute onset DCM in a child admitted to a referral hospital in Jakarta, Indonesia. With the absence of isolation from the cardiac muscle, the role of rhinovirus in the pathogenesis is not definite. Further studies with more comprehensive diagnostic evaluation of myocarditis, with extensive viral testing panels and antibody assays, are needed to confirm the association, as well as advanced characterization of the virus to determine any amino acid mutations with the potential to cause cardiac complications.
